# Departure of Professor Brainard

**Published:** 1853-11

**Authors:** 


*Departure of Professor Brainard.*

PROFESSOR BRAINARD sailed for Europe on the 15th ult. He expects to spend the winter in Paris, for the purpose of becoming acquainted with the Physicians and Surgeons of that city; and also for observing the present position and progressive tendencies of that department of our science, to which he has devoted himself with such unwearied industry, and with such signal success.

We are gratified in being able to announce to our readers a series of letters from Prof. B., giving his impression of men and things in Europe.

He will return in May next, to resume the practice of Surgery in this city. J.

